# Experimental Study on Wound Area Measurement with Mobile Devices

**DOI:** 10.3390/s21175762

**Published:** 2021-08-26

**Authors:** Filipe Ferreira, Ivan Miguel Pires, Vasco Ponciano, Mónica Costa, María Vanessa Villasana, Nuno M. Garcia, Eftim Zdravevski, Petre Lameski, Ivan Chorbev, Martin Mihajlov, Vladimir Trajkovik

**Affiliations:** 1 R&D Unit in Digital Services, Applications, and Content, Polytechnic Institute of Castelo Branco, 6000-767 Castelo Branco, Portugal; filipemiguel2801@gmail.com (F.F.); vasco.ponciano@ipcbcampus.pt (V.P.); monicac@ipcb.pt (M.C.); 2 Instituto de Telecomunicações, Universidade da Beira Interior, 6200-001 Covilhã, Portugal; ngarcia@di.ubi.pt; 3 Global Delivery Center (GDC), Altranportugal, 1990-096 Lisbon, Portugal; 4 Centro Hospitalar do Baixo Vouga, 3810-164 Aveiro, Portugal; 72152@chbv.min-saude.pt; 5 Faculty of Computer Science and Engineering, University Ss Cyril and Methodius, 1000 Skopje, North Macedonia; eftim.zdravevski@finki.ukim.mk (E.Z.); petre.lameski@finki.ukim.mk (P.L.); ivan.chorbev@finki.ukim.mk (I.C.); trvlado@finki.ukim.mk (V.T.); 6 Laboratory for Open Systems and Networks, Jozef Stefan Institute, 1000 Ljubljana, Slovenia; martin@e5.ijs.si

**Keywords:** wound area measurement, mobile application, segmentation, threshold, image processing techniques

## Abstract

Healthcare treatments might benefit from advances in artificial intelligence and technological equipment such as smartphones and smartwatches. The presence of cameras in these devices with increasingly robust and precise pattern recognition techniques can facilitate the estimation of the wound area and other telemedicine measurements. Currently, telemedicine is vital to the maintenance of the quality of the treatments remotely. This study proposes a method for measuring the wound area with mobile devices. The proposed approach relies on a multi-step process consisting of image capture, conversion to grayscale, blurring, application of a threshold with segmentation, identification of the wound part, dilation and erosion of the detected wound section, identification of accurate data related to the image, and measurement of the wound area. The proposed method was implemented with the OpenCV framework. Thus, it is a solution for healthcare systems by which to investigate and treat people with skin-related diseases. The proof-of-concept was performed with a static dataset of camera images on a desktop computer. After we validated the approach’s feasibility, we implemented the method in a mobile application that allows for communication between patients, caregivers, and healthcare professionals.

## 1. Introduction

Digital cameras and cameras embedded in mobile devices increase the quality and resolution of the images captured [1,2], allowing for powerful solutions for different areas of human life [3,4]. Currently, the emergence of studies related to healthcare and technology is increasing, and it is participating in the new era of medicine related to patient empowerment [5]. Patient empowerment is a concept that gives methods and equipment at low cost to patients to help them take care of their health in remote contact with their healthcare professional [6,7].

Several healthcare solutions may be handled with technological equipment, including the measurement of a wound area to control its healing evolution, which is vital in infected and chronic wounds [8]. However, the cost of these solutions is essential to the widespread acceptance and dispersion of the developed solutions [9]. The use of standard mobile devices with different cameras allows for less invasive and low-cost solutions [10]. However, various studies related to wound area measurement with mobile devices have produced no standard and validated solutions that are available on the market [11,12]. Furthermore, the availability and acceptance of these solutions are difficult to achieve because they need medical validation and approval by the regulatory entities to be used by healthcare professionals and hospitals [13,14].

The main reason for this study is the development of a valid and reliable solution for the measurement of the wound area to reduce the costs in the measurement of the wound size and the monitoring of the speed of the wound healing [15,16]. Thus, it may be a factor in other healthcare problems, especially in older adults and people with chronic diseases [17].

Different computer vision frameworks are also available for mobile devices. However, this study’s primary purpose consists of developing a method for accurate wound measurement with desktop and mobile devices. The developed method includes different stages, including image capture, conversion to grayscale, blurring, application of a threshold with segmentation, identifying the wound part, performing dilation and erosion of the detected part, identifying accurate data related to the image, and measuring the wound area. Furthermore, the method was implemented in a mobile application that includes other functionalities, including storing different pictures, automatic measurement of the wound area, and contact between patients and healthcare professionals. Furthermore, the ease of developing these solutions is another advantage due to the growing number of libraries and frameworks that allow for faster growth. Finally, the size and ease of use are unique opportunities to build solutions that help healthcare professionals follow-up with patients and diseases.

The developed method was implemented with the OpenCV framework [18], including the techniques needed to achieve this study’s primary goal. OpenCV contains multiple image processing algorithms with scaling invariance, which has been successfully applied for contour detection and segmentation under variable light conditions [19,20]. These properties are essential so that the patient can remotely take a photo and send it to the healthcare professional with all the wound’s characteristics, making it unnecessary to travel to a consultation. Furthermore, the method now presents itself as an enormous advantage since, with the pandemic that the world is experiencing, the gathering of people may be a factor in the emergence and increase of contagions. The method was developed with the supervision of healthcare professionals. Other features that will be useful to implement in the future are related to the color of the wound, which allows for the identification of its state, and specific problems with the wound analyzed. Furthermore, as the resolution of the photos is better controlled with a mobile device, the conversions between pixels and metric units are more accurate with a mobile device. Still, successfully applying such algorithms in a scalable fashion requires distributed architectures relying on Apache Spark [21].

The remainder of this paper is organized as follows. Section 2 presents an overview of the state-of-the-art. Section 3 describes the different parts of the method that was implemented. Section 4 presents the results with the implementation of the desktop application, and the implementation of the same techniques in a mobile application is presented in Section 5. Finally, Section 6 discusses the results based on the healthcare professional’s analysis and concludes this paper.

## 2. Related Work

Several studies applied the capabilities of mobile devices and related systems, such as systems that use the paradigm of the IoT, to most different areas, such as mobility and healthcare. In this article, the authors perform many tasks to identify the structure, the area, and other features related to the wound. The most critical components approached in this study are the saturation, the inversion and filtering of saturation values, and the performance of segmentation with the threshold. Previously, the authors analyzed several studies to measure the wound area [22]. Therefore, many studies were analyzed to gain insight into the best approaches to measuring the wound area. Next, they proposed a new method based on the literature that contains different stages to measure the wound area [23]. It is composed of capturing the image, converting the image to grayscale, applying a threshold to enhance the image’s contrast with Otsu’s thresholding algorithm, performing segmentation with the threshold, finding the contours of the wound, and measuring the wound area as the number of pixels.

In [24], the authors conduct a study on detecting a wound on the skin of a human patient. In the initial stage, the authors use marks to identify the position of an injury on a patient. Then, the image is converted to grayscale. Next, other features are calculated and used to develop a method that includes such processes as dimensionality reduction by finding an orthogonal projection of the high-dimensional data into a lower-dimensional subspace. Finally, the implementation finishes with median filtering, a threshold to enhance the image’s contrast with Otsu’s thresholding algorithm, the erosion operation on the binarized image, and suppression of the structures connected to the image border dilation operation.

In [25], the authors convert an image using HSV to perform the segmentation of a wound. Afterward, they extract the saturation space and the color space and implement two approaches: a threshold and image contrast with Otsu’s thresholding algorithm. In the end, the authors calculate the number of black pixels to design a plot healing curve.

The authors begin correcting and calibrating the color in 2D images to measure the wound area. Then, reconstruction and segmentation using computer techniques are performed. In the end, the design, the area, the number of pixels, and the size of the wounds are calculated [26]. The tuning of hyper-parameters could be improved by fine-tuning the adaptive learning rates and label smoothing [27]. This is particularly important in remote medicine applications where validation is difficult and time consuming [28].

The authors intend to define the toroidal geometry using images of wounds segmented and located in the ulcer region [29]. After that, the image is decomposed using different contrast levels and the threshold technique, and Otsu’s algorithm is used to detect the contours of the wound. Other techniques for manipulating an image are grayscale techniques, linear combinations of discrete Gaussians (LCDG), and minimizing the noise with a Generalized Gauss–Markov Random Field (GGMRF) image model calculation of the area of the wounds.

In [30], the authors began with extracting RGB channels and the blue channel from images. After that, the conversion from RGB to grayscale was performed. In the end, a low-pass filter, a threshold, the degree intensity, Otsu’s algorithm, and the support vector machine were used to classify and identify all the features present in the image of a wound.

The authors intend to calculate and identify many features related to a wound [31]. The authors start with the 3D reconstruction of the image. After that, the authors evaluate many features, such as the surface and the object, by a dense point cloud. Next, the authors use histogram equalization using Otsu’s thresholding algorithm. Finally, the authors measure the induration of a margin.

In [32], the authors used the OpenCV library to identify and calculate a wound area. The authors used a segmentation and detection algorithm, applied the grabCut algorithm, found the intensity values of colors with an OpenCV histogram, and implemented the coin detection algorithm to enhance the wound area’s measurement.

The authors of [33] implemented a scaling method for measuring the wound size by placing a rectangular box in the image with a mobile device.

In different studies, we have found other methods implemented with a mobile application. For example, the authors of [34] allowed for remote wound measurement, tracking, and diagnosis. They started with the computation of the absolute scale of the wound with optics equations. However, the pictures must be captured at a distance of 20 cm.

In [35], the authors implemented a method for measuring the wound area based on segmentation techniques, the camera distance, the camera angle, and lighting conditions. First, they cropped the center of the wound and removed unnecessary artifacts. After that, they extracted the saturation plan of the HSV color model to identify the contour based on the contrast between regions and applied a Gaussian filter. Finally, they transformed the image to grayscale, segmented it, designed the outline, and measured the wound area.

To measure a surgical wound’s size, the authors of [36] used the HSV color model to implement normalization techniques and the SEED algorithm for superpixel segmentation.

The authors of [37] measured the wound area by implementing the Enhance Local Contrast (CLAHE) algorithm. First, they performed white balance, anti-glare, and Contrast-Limited Adaptive Histogram Equalization procedures. Next, the authors used two algorithms, the level set and snake model algorithms, to perform segmentation.

Other studies have been conducted on wound area measurement [38,39,40,41], but the methods implemented were the same as in the previously described studies. Taken together, the studies reveal that wound area measurement is an active and vital subject and that correct measurement is crucial in order to avoid future problems. Furthermore, in 2020, the challenges associated with the COVID-19 pandemic opened the door to remote work [42,43] and remote medical consultations, where these measurements are essential.

## 3. Methodology

### 3.1. Dataset

**Figure 1:**
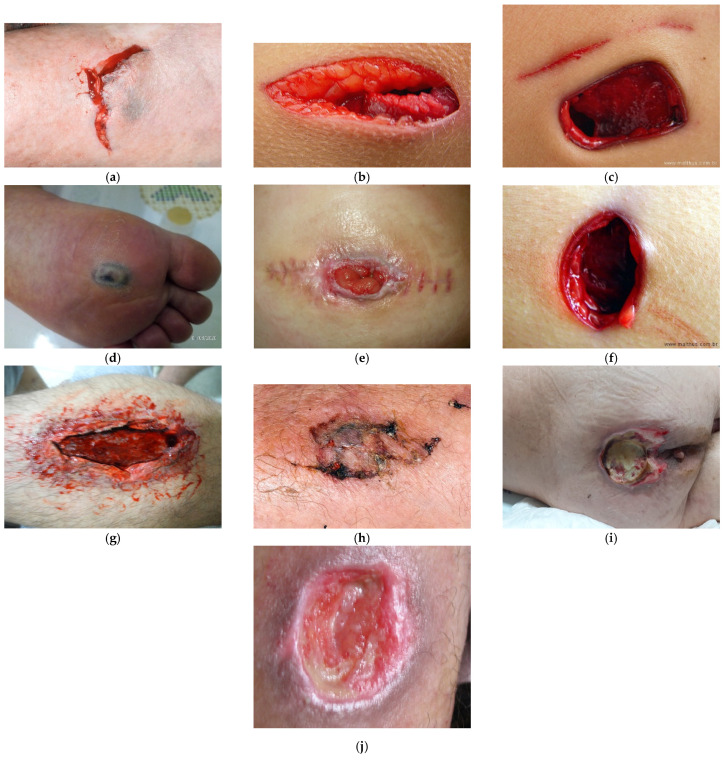
Example images of wounds. (**a**)—wound image in a human arm [44]; (**b**)—wound image in a human trunk; (**c**)— wound image in a human trunk; (**d**)—wound image in a human foot; (**e**)— wound image in a human trunk [45]; (**f**)— wound image in a human trunk [45]; (**g**)—wound image in a human arm [45]; (**h**)—wound image in a human arm [45]; (**i**)—wound image in a human tail; (**j**)—wound image in a human leg [44].

To estimate the wound size in various images, as shown in Figure 1, we asked different authors about the pictures they used for further comparison (see Figure 1b,c). Additionally, we collected other images from other databases, including Science Photo Library [44] (see Figure 1a,j) and iStockPhoto [45] (see Figure 1e–h), and other images previously acquired by professionals working at the School of Health at the Polytechnic Institute of Castelo Branco in Portugal (see Figure 1d,i). The used dataset contains images of real-life wounds that were captured from patients from different countries. The dataset includes ten images captured using different kinds of devices. The photos have the same resolution and the same Dots Per Inch (DPI) properties, i.e., 300 dpi.

### 3.2. Data Processing Steps

**Figure 2:**
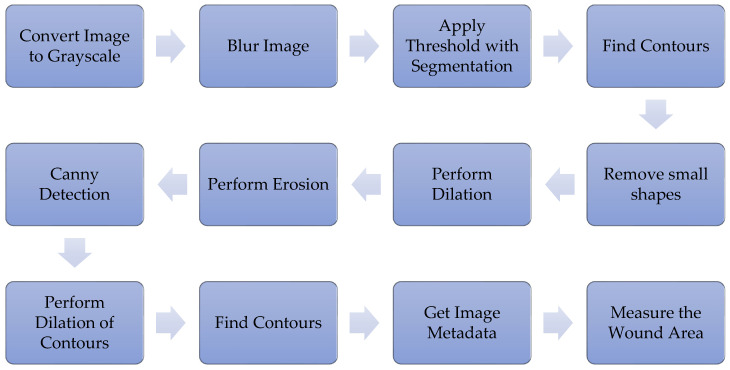
Data processing steps.

Based on the literature on image processing tasks that involve object identification and segmentation, we identified the key steps that need to be taken for successful wound size estimation. First, a sequence of techniques was selected to measure the wound area. Of course, the selection always depends on the quality of the image, including the conditions of the image’s acquisition, the quality of the camera, and the techniques applied. Figure 2 shows the sequence of methods used to measure the wound area that was proposed in [23]. One additional preprocessing procedure that was performed was the standardization of the DPI of all images.

**Figure 3:**
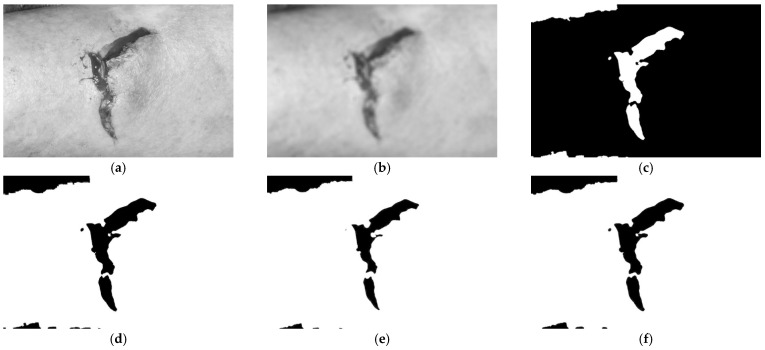

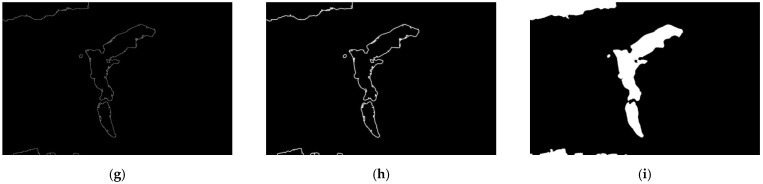
Wound image processing steps. (**a**)—wound image after conversion to grayscale; (**b**)—wound image after blurring; (**c**)—wound image after application of threshold and segmentation techniques; (**d**)—wound image after contour detection; Figure (**e**)—wound image after dilation of the white region; (**f**)—wound image after erosion of the white region; Figure (**g**)—wound image contours after canny detection; (**h**)—wound image contours after dilation; (**i**)—final wound image.

The remainder of this section describes the specific image processing steps of the proposed approach. First, an illustration of these image processing steps and their impact on the image is shown in Figure 3 in the Results section. Then, the different libraries used to implement the proposed method in desktop and mobile devices are described in Section 3.3. Finally, a mobile application with the presented measurements is described in Section 3.4.

#### 3.2.1. Convert the Image to Grayscale

Initially, an image containing a wound was selected in order to start applying the sequence of proposed techniques. The image’s conversion to grayscale consists of converting the original colors in the RGB color space to grayscale [46,47] in order to identify the wound region better. The method must be adapted to desktop and mobile devices, and the OpenCV framework [18] is already prepared for this purpose. After the conversion to grayscale, blurring techniques must be applied.

Based on the OpenCV library, Figure 1a was read and decoded into a binary structure. Next, the function *Imgproc.cvtColor* was used with the parameter Imgproc.COLOR_BGR2GRAY, which converts the image to different levels of gray. The results obtained are presented in Figure 3a.

#### 3.2.2. Blur the Image

Next, for the measurement of the wound, some parts of the image must be ignored. Thus, as presented in other studies [25,48], the image must be blurred to ignore particular objects and only detect the wound region. In addition, the method must include the correct definition of the blur parameters. Finally, after the accurate blurring of the image, we implement thresholding techniques to perform wound segmentation. The results obtained are presented in Figure 3b.

#### 3.2.3. Threshold with Segmentation

Sequentially, a threshold with segmentation will binarize the image, transforming the pixels with high intensity to black and the pixels with a lower intensity to white [25,49]. Thus, this technique will create a binarized image with black and white, where the wound will be black and the surrounding background will be white. For better identification of the wound, the related region must be white. Thus, the binary image must be inverted using the functions available in the OpenCV framework [18]. The results obtained are presented in Figure 3c. Next, we find contours during the preliminary stage of the identification of the wound.

#### 3.2.4. Find Contours

Preliminarily, the wound’s contours can be found by identifying the limits between the different colors present in the binarized image [25,48] using the proper functions available in the OpenCV framework [18]. The identified contours include those unrelated to a wound, where the small contours are considered as incorrect contours that are removed during the next stage.

#### 3.2.5. Remove Small Shapes

Small shapes can be considered to be noise elements, and they must be removed before the wound area can be correctly measured [50]. It is also possible to use the different functions available in the OpenCV framework [18]. The results obtained are presented in Figure 3d. However, some minor contours are close to the wound area and are a part of it. For this case, the dilation method is needed.

#### 3.2.6. Perform Dilation for Canny Detection

The small shapes found must be merged with the large shape, which is considered to be a wound. Thus, all of the different shapes are dilated to create an oversized shape. The results obtained are presented in Figure 3e. Then, as it is not the real wound area, erosion must be performed.

#### 3.2.7. Perform Erosion for Canny Detection

The measured area must approximate as closely as possible the real wound area. Thus, erosion must be performed with the same size as used in the dilation process to maintain the joint shapes and adjust the size of the large shape. The results obtained are presented in Figure 3f.

#### 3.2.8. Canny Detection

Next, the contours of the shape in the picture can be correctly detected with the canny detection method, which is available in the OpenCV framework [18]. After detection is performed, the contours are defined in the image. The results obtained are presented in Figure 3g.

#### 3.2.9. Perform Dilation to Find Contours

The dilation method is also applied to merge the small contours in the image to improve the measurement of the wound area. The results obtained are presented in Figure 3h. However, to detect the actual wound area, different characteristics of the original image must be known.

#### 3.2.10. Obtain Image Metadata

The Commons Imaging framework [51], previously called the SanseLan framework, can obtain different parameters related to the wound image, including the image’s width, the image’s height, and the dots per inch (DPI) related to the image’s width and the image’s height. Therefore, it allows us to adapt the measurement of the real wound area using the different conversions between pixels and centimeters based on the image’s DPI.

#### 3.2.11. Measure the Wound Area

The OpenCV framework [18] includes a method for measuring the area between the defined contours in Section 3.2.9. However, the wound area’s measurement includes different challenges, one of which is the image’s resolution, including the DPI. For example, each picture has its width and height defined in pixels and retrieved by the Commons Imaging framework [51]. However, the healthcare professional needs to measure these values in metric units, such as centimeters, to control the healing evolution. Furthermore, the images can be taken with different cameras at different resolutions. Thus, the implemented method needs to consider Equation (1), and, based on the image’s metadata, the width and height values must be converted to centimeters.

(1) $${\mathrm{value}}_{\mathrm{cm}}={\mathrm{value}}_{\mathrm{px}}\times \left(2.54÷\mathrm{DPI}\right)$$

Based on the conversion of the total width and the total height of the image to centimeters, the total area of the image can be obtained. It must be done with the values in centimeters and the values in pixels. This value can be converted by comparing the different areas previously measured with the retrieved wound area. Thus, the wound area is measured and defined as in Figure 3i.

### 3.3. Evaluation Criteria

Considering the dataset used, we asked different authors about the correct size of the images. Unfortunately, they only answered with the correct DPI values for the images, confirming that the correct value is 300 dpi for all images. Thus, considering the high capabilities of desktop computers compared with mobile devices, the desktop computer’s measured value was regarded as a baseline for the comparison with the mobile application when applying cross-validation techniques and measuring the mean absolute error (MAE).

### 3.4. Mobile Application

The second part of this study consists of creating a mobile application that includes the developed method for measuring the wound area. This mobile application was developed with three types of users in mind: patients, caregivers, and healthcare professionals.

If the logged-in user is a patient, the mobile application allows the user to change their personal data, take a picture of a wound using the camera, obtain a picture of a wound from the gallery, and check the list of uploaded images.

If the logged-in user is a caregiver, the mobile application allows the user to change their personal data and see the list of patients. The caregiver can select a patient and perform the same actions as the patient, such as change the patient’s data, take a picture using the camera, obtain from the gallery a picture of a wound, and check the list of uploaded images of the selected patient.

If the logged-in user is a healthcare professional, the mobile application allows the user to change their personal data and see the list of patients. However, when the healthcare professional selects a patient in the list, the doctor will change the patient’s data and see the selected patient’s pictures. In addition, the healthcare professional can choose a specific photo, and, after that, the healthcare professional can delete an image used to analyze a wound with the previously presented method. In the end, an estimation of the wound area is returned to the healthcare professional. Finally, the mobile application allows for the communication of the results to the patient.

## 4. Results

The algorithm presented in this study consists of an experimental algorithm that uses the OpenCV framework and other image processing techniques that measure the wound area over time to check for possible pathologies associated with the development of skin-related diseases. First, the image is passed through to the Java-developed program, which runs with the NetBeans IDE. The method has the following steps (based on Section 3 and Figure 2) and is illustrated in Figure 3.

### 4.1. Convert the Image to Grayscale

Based on the OpenCV library, the image shown in Figure 2 was read and decoded into a binary structure. Next, the function *Imgproc.cvtColor* was used with the parameter *Imgproc.COLOR_BGR2GRAY*, which converts the image to different levels of gray. The results obtained are presented in Figure 3a.

### 4.2. Blur the Image

Next, the function *Imgproc.blur* was applied to the image shown in Figure 3 to smooth the image, which has a grid size of 19 × 19. The results obtained are presented in Figure 3b.

### 4.3. Apply the Threshold with Segmentation

Next, Otsu’s algorithm is applied. For this process, the function *Imgproc.threshold* converts the image into only white and black colors with the parameter *Imgproc.THRESH_OTSU*. After that, the colors of the image were inverted with *Core.bitwise_not,* resulting in a wound region with a white color and a black background as presented in Figure 3c.

### 4.4. Find Contours and Remove Small Shapes

The primary objective of this method is to detect the contours of the wound using the color contrast, which allows us to differentiate the area of the image where the wound is located. This operation is done with the function *Imgproc.findContours*, which fills a structure with contours, where the close shapes are searched for with the parameter *Imgproc.CHAIN_APPROX_SIMPLE* and the mode *Imgproc.RETR_LIST*, which allows us to detect all contours, including the inner and outer circumference contours; however, the detected contours do not establish the level. After detecting the different shapes, their areas were measured with the function *Imgproc.contourArea*, and those outlines with an area less than a defined value were removed. Finally, the final image was defined with the remaining objects of the contours in black on a white background. The function *Imgproc.circle* was used to fill the small regions of the area with the parameter *Core.FILLED*. The result obtained is presented in Figure 3d.

### 4.5. Perform Dilation

Once the contours of the image were found, small regions between the different shapes were avoided. However, these regions are a part of the wound area. Thus, the function *Imgproc.dilate* was applied with an ellipsis size of 11 × 11 in the white area. The result is shown in Figure 3e.

### 4.6. Perform Erosion

Once the image is dilated, the region of the wound will be highlighted. Next, to reconstruct the wound, the white part of the image was eroded using the function *Imgproc.erode* with an ellipsis size of 11 × 11. The result is shown in Figure 3f.

### 4.7. Canny Detection

Once the image is eroded, the region of the wound is expected to be represented. The canny detection method was applied to design one picture only with the contours of the wound as presented in Figure 3g.

### 4.8. Perform Dilation of the Contours

Once the contours are defined, the different regions of the wound can be identified. Next, the dilation of the shapes was performed for the correct measurement of the inside area as presented in Figure 3h.

### 4.9. Find Contours, Obtain Image Metadata, and Measure the Wound Area

Once the dilated contours are defined, the function *Imgproc.findContours* was applied to search for the close elements using *Imgproc.CHAIN_APPROX_SIMPLE* and the *Imgproc.RETR_LIST* mode, which allows us to detect all contours, including inner circumference and outer shape contours. After the detection of all contours, the outlines that were smaller than 50 were discarded. Thus, the final joint contours are shown in Figure 3i.

### 4.10. Measurement of the Wound Area

Using the contours on the final processed image (see Figure 3i), the average of all contours was calculated to exclude the small shapes below the average. In addition, the function *Imgproc.contourArea* was applied to sum the size of the areas of the large outlines in pixels, resulting in 54,746.5 px. Next, the image metadata were retrieved for the correct measurement of the wound area, including the resolution of the image in DPI and the width and height of the picture. Based on Figure 3i, the values obtained are presented in Table 1.

The image metadata will be used to convert the measured area in pixels to a metric unit. Equation (1) was applied to convert the width and height values presented in Table 1 from pixels to centimeters, where value_cm_ represents the converted values in centimeters, and value_px_ represents the values in pixels. The converted values and areas in cm^2^ are presented in Table 2, which shows that the equivalent wound area in cm^2^ is 3.9254 cm^2^.

### 4.11. Summary of the Analysis of the Dataset with the Desktop Application

Based on the implementation of the methods presented in Section 4.1, Section 4.2, Section 4.3, Section 4.4, Section 4.5, Section 4.6, Section 4.7, Section 4.8, Section 4.9 and Section 4.10 for Figure 1a, the same techniques were implemented for Figure 1b–j. The results are shown in Table 3.

## 5. Mobile Application

### 5.1. Description of the Functionalities

Regarding the results presented in Section 4, a mobile application was developed to promote the measurement of the wound area. It is vital to exploit the use of telemedicine and to promote remote contact between patients and healthcare professionals.

**Figure 4:**
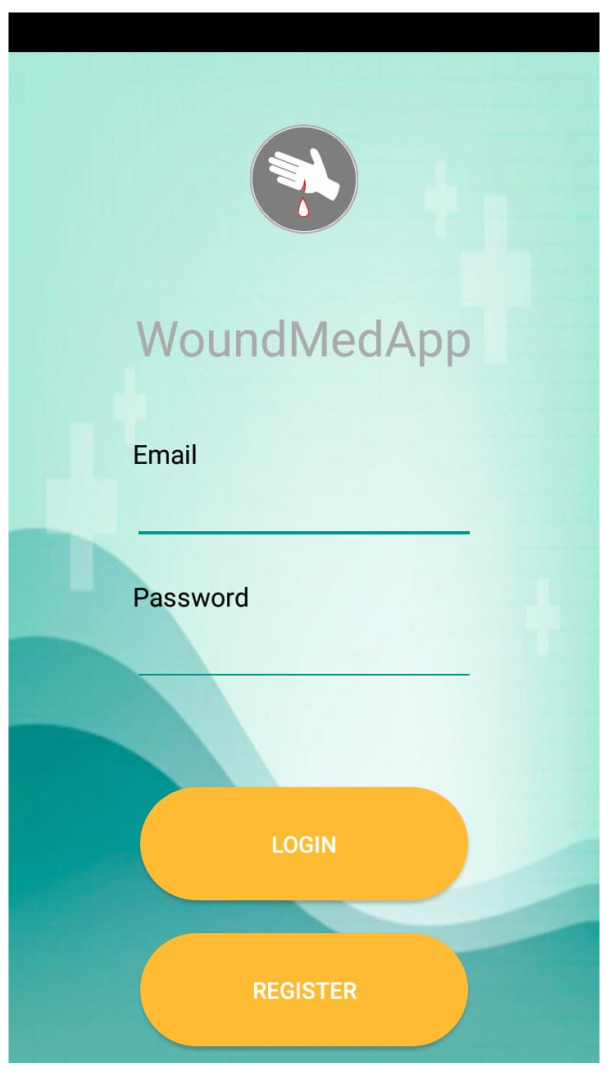
Sign In.

**Figure 5:**
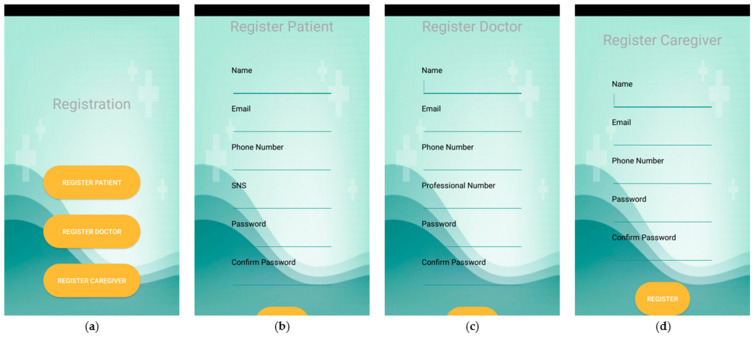
Sign Up. (**a**) shows the registration menu that allows for the registration of a patient in (**b**), the registration of a doctor in (**c**), and the registration of a caregiver in (**d**).

As presented in Section 3.2, the mobile application considers three types of users with different functionalities. All users have the option to sign in (Figure 4) and sign up with varying levels of access (Figure 5) to the mobile application.

**Figure 6:**
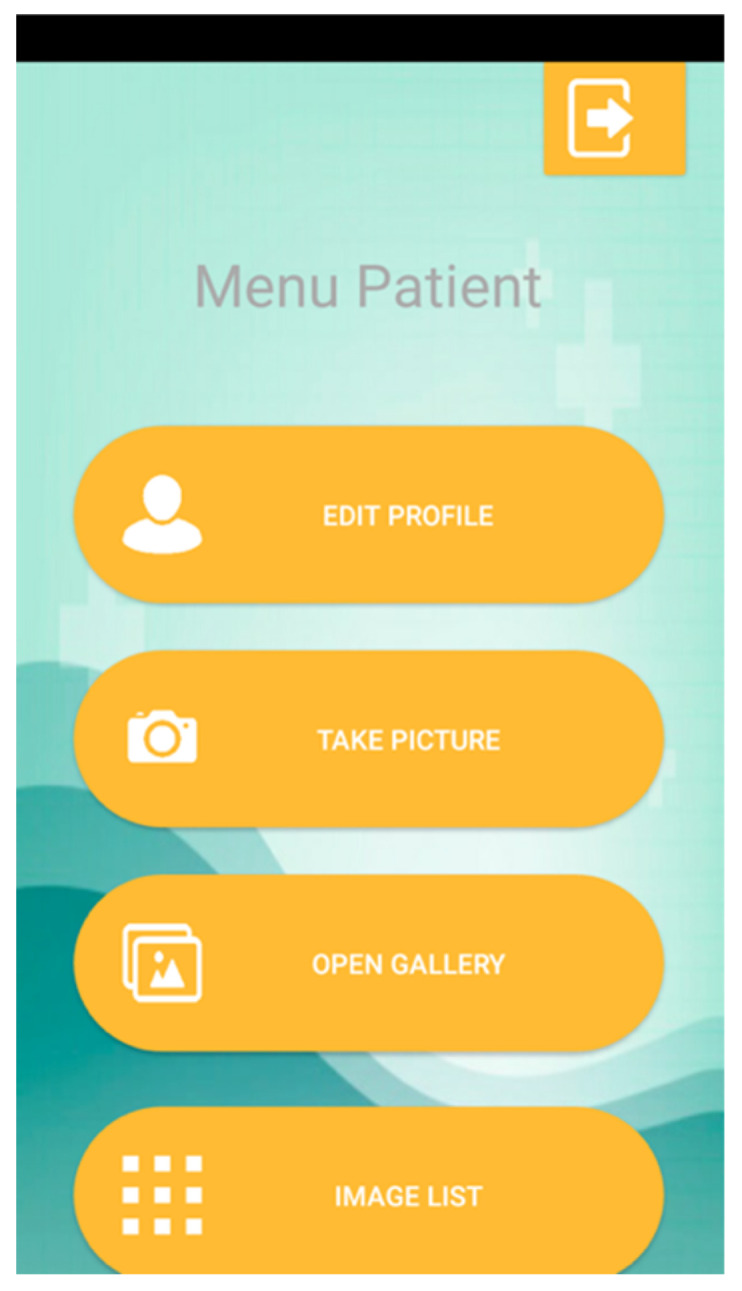
Patient’s Menu.

Now, each type of user has particular actions available to them. Firstly, a patient can access a menu with four options (Figure 6): update your data (with a similar interface to that for registration); take a photo; select a picture from the gallery; and check the uploaded images.

**Figure 7:**
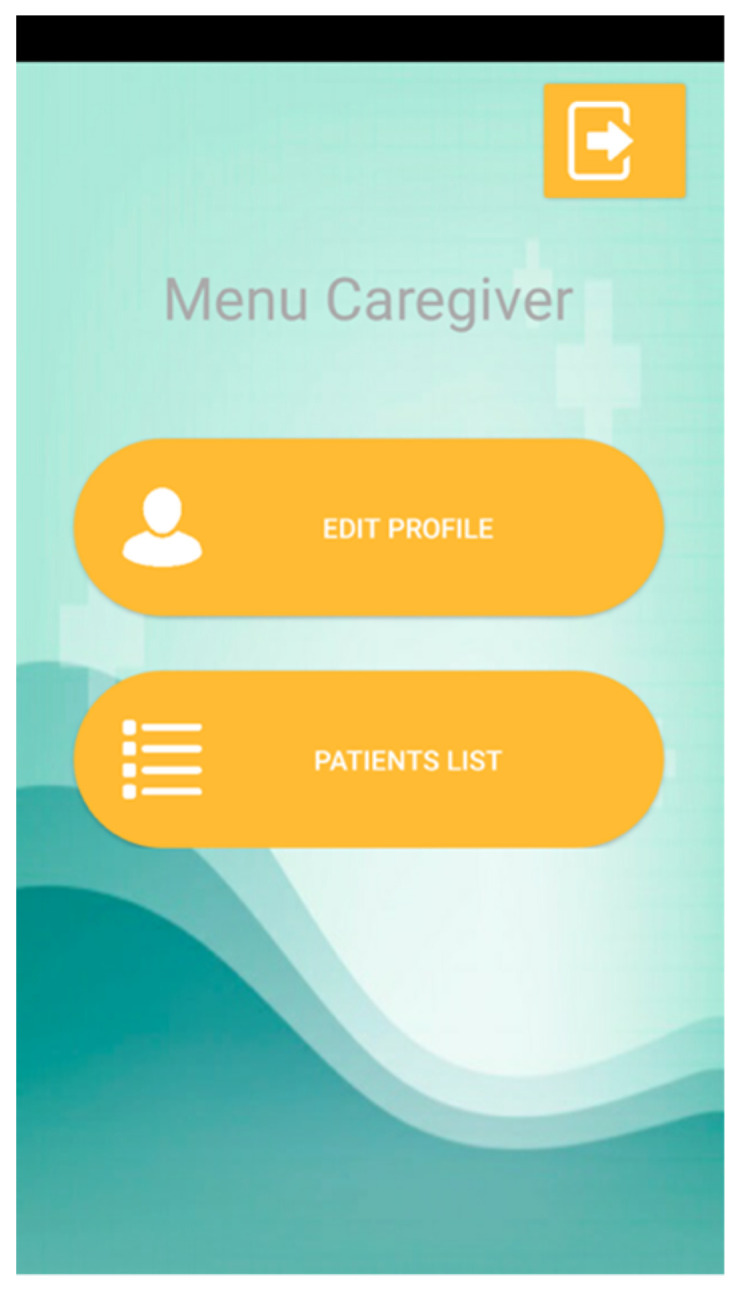
Caregiver’s Menu.

Next, a caregiver can access a menu with two options (Figure 7): update your data (with a similar interface to that for registration); and list patients. The list of patients allows the caregiver to access a contextualized menu identical to that shown in Figure 6.

**Figure 8:**
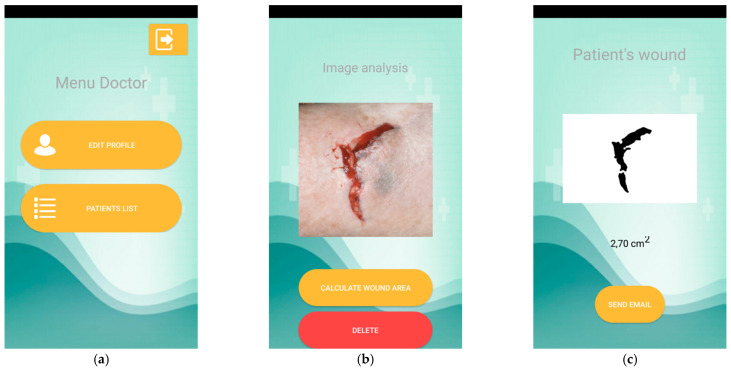
Doctor’s actions. (**a**) shows the doctor’s menu. (**b**) shows the details of a selected image in the list. (**c**) shows the results of the wound analysis.

Finally, a healthcare professional can access a menu with two options (Figure 8a): update your data (with a similar interface to that for registration); and list patients. The list of patients allows the healthcare professional to edit patient data and, based on the image list, can access the interface for a specific image and perform different actions, including ‘delete this image’ and ‘analyze this image’, as presented in Figure 8b. Figure 8c shows the results of the wound analysis, the final image with the contours filled that is equivalent to Figure 3i, and that the measured wound area equals 2.70 cm^2^. Additionally, the healthcare professional can contact patients directly to inform them about their wound’s evolution.

### 5.2. Summary of the Analysis of the Dataset with the Mobile Application

Based on the implementation of the mobile application presented in Section 4.1 for Figure 1a, it was also implemented for Figure 1b–j. The results are shown in Table 4.

## 6. Discussion

The measurement of the real wound area is not easy to perform because it requires us to consider the quality of the image, the total number of pixels in the picture, and the number of pixels that is to be considered a wound, and the method must be able to convert the values to metric units. Sometimes, uploading an image to a mobile application or sending it by other electronic methods causes a loss of some of the image’s metadata. Thus, the wound area cannot be correctly measured because the quality of the image or the DPI does not allow the method to obtain the real area. Moreover, another problem is the distance between the camera and the wound surface. Proximity sensors have low accuracy with respect to the detection of the distance between the surface and the camera. Additionally, the distance was not considered when measuring the wound area in order to allow the method to be implemented in desktop devices.

Based on a comparison of the measurement of the wound areas by the desktop application (see Table 3) and the mobile application (see Table 4), the differences in the areas are presented in Table 5. The mean absolute error (MAE) was calculated based on these values and equals 34.7%.

Based on the MAE, the adjusted values of the wound area measured using the method implemented in the mobile application are presented in Table 6. Based on the reported values, the MAE was calculated and equals 21.5%. Additionally, it was proved that higher MAE values are related to more than one wound zone (see Figure 1c,f), more elements or unrelated zones (see Figure 1d,g,j), and wounds in the late healing stage (see Figure 1h). Furthermore, the MAE is also related to the luminosity of the images and the resolution of the photos. However, for those pictures that have reliable quality, the MAE is, on average, 4.4%.

Regarding the images used for the testing of the algorithm, we found some problems related to luminosity and the presence of other artifacts. These are the two significant problems associated with implementing the algorithm that were found in other studies [35]. However, in some cases, we reported an error without calibration marks that was lower than that of the method implemented in [24].

The methods presented by the authors of [29,30,36,38] need high processing power and cannot be implemented in mobile devices. They provide only theoretical studies that do not provide contact between patients and healthcare professionals.

The most similar studies to our work found in the literature are [25,26,31,33]. However, we have made it possible for three types of users to use the mobile application and for patients to provide feedback to healthcare professionals. Similarly, the mobile application presented by Wu et al. [32] does not allow for contact between patients and healthcare professionals; however, they studied the use of calibration marks. Yee et al. [34] enable communication between healthcare professionals and patients, but they acquire video that may have some network constraints in rural areas. Sequentially, the authors of [39,40,41] require more complex conditions for the image’s acquisition with the mobile application and a specific device is required. Finally, Huang et al. [37] implement a method in a mobile device that does not allow for contact between healthcare professionals and patients and they use an outdated platform, i.e., a Windows phone.

Regarding the different constraints presented in the literature, our mobile application allows for monitoring by healthcare professionals and contact with their patients. However, it is only a prototype that remains in the process of being tested and validated by our team of healthcare professionals and may become available on the market in the coming months. The results provided by our app are exciting, but problems related to the distance between the camera and the mobile device remain in the process of being solved.

Based on the studies analyzed, using a mobile device is more practical, and most of the recent studies use a mobile device. Nursing and healthcare professionals urgently need to monitor their patients during the COVID-19 pandemic.

Most of the images used in the different studies were not made available for testing and comparison with the results of other authors. However, we asked various authors to share multiple photos for further testing, but we received no answer. Therefore, we consider that the areas retrieved using the pictures included in our tests provided realistic values.

The developed mobile application facilitates more accessible communication between healthcare professionals and patients. However, we also consider that there may be patients without the capacity to use a mobile device. Therefore, a caregiver can be authorized to capture and upload the images on behalf of the patient.

Regarding the studies analyzed, only 29% of them included collaboration with medical professionals to develop and validate the proposed method. Wounds are always different, and their treatment will not always be the same because it depends on each person. In medical science, it is difficult to develop intelligent methods without the supervision of healthcare professionals. However, it is currently difficult to measure the evolution of the healing of a wound, and the developed mobile application allows for the monitoring of the treatment remotely. Other healthcare professionals and nursing professionals who were not a part of the team were contacted to evaluate the mobile application’s performance. They said that it would be helpful for the community and that it facilitated their work.

An important consideration when estimating the wound size in an image is related to the DPI of the image. Some libraries may, in some cases, incorrectly infer the DPI of a particular image and, consequently, produce a very incorrect wound size, even though the contour of the wound was very accurately identified. Therefore, a mandatory preprocessing step is to standardize the DPI of all images and ensure that the subsequent processing steps shown in Figure 2 use the correct DPI.

One limitation of the approach is that the image must always be captured with the same distance between the mobile device and the patient. Even though this is a limiting factor, it could be improved in the future. One idea is to place an object with a constant size (e.g., a coin) in the frame next to the wound to serve as a reference. This would help the method to identify the wound’s scale with respect to the object with a constant size. Another idea is to use a constant distance from the lens to the wound, again with an object with a consistent size. For example, a book with a standard cover size (e.g., an A5 book’s cover size is 210 × 148 mm) could be used as a measuring device or as a tripod on which the mobile device is to be placed to take a picture of the wound.

## 7. Conclusions

This paper shows the implementation of a method for measuring a wound area using different image processing techniques. The developed method was tested with images from medical databases and other pictures retrieved from local healthcare centers. The conditions under which the images were taken and the quality of the digital cameras and built-in mobile device cameras were found to influence the results.

Based on the case study presented in Section 4 and Section 5, the method implemented in a desktop machine retrieved a higher error value for the wound area than the same method implemented in a mobile device. Therefore, it is expected that the mobile device will provide a more reliable estimation of the area because it identifies and discards a zone in a later healing stage.

In future work, the method must be tested with more images. Furthermore, notifications must be added to the mobile application in order to notify the healthcare professional when the patient has uploaded a new photo or a caregiver has uploaded an image related to a specific patient. In addition, the relationship between the patient, the caregiver, and the healthcare professional must be added. The mobile application should be tested further with medical healthcare professionals in real cases to improve its usability.

## Figures and Tables

**Table 1:** Image metadata.

Parameters	Values
Physical Width (DPI)	300
Physical Height (DPI)	300
Width (px)	1024
Height (px)	706
Total area (px)	749,772

**Table 2:** Final measured values.

Parameters	Values
Width (px)	1024
Height (px)	706
Width (cm^2^)	8.99
Height (cm^2^)	5.98
Total area (px)	749,772
Total area (cm^2^)	53.7602
Wound area (px)	54,746.5
Wound area (cm^2^)	3.9254

**Table 3:** Wound area measured by a desktop application.

Figure	Wound Area Measured by Desktop Application (cm^2^)
1a	3.92
1b	8.56
1c	7.52
1d	4.98
1e	3.50
1f	7.07
1g	6.81
1h	1.74
1i	4.58
1j	2.27

**Table 4:** Wound area measured by the mobile application.

Figure	Wound Area Measured by a Mobile Application (cm^2^)
1a	2.70
1b	6.50
1c	6.69
1d	2.52
1e	2.06
1f	5.95
1g	2.91
1h	0.83
1i	3.20
1j	1.16

**Table 5:** Comparison of the wound area measured by the different platforms.

Figure	Wound Area Measured by a Desktop Application (cm^2^)	Wound Area Measured by a Mobile Application (cm^2^)	Difference (cm^2^)
1a	3.92	2.70	−1.22 (−31.1%)
1b	8.56	6.50	−2.06 (−24.0%)
1c	7.52	6.69	−0.83 (−11.0%)
1d	4.98	2.52	−2.46 (−49.4%)
1e	3.50	2.06	−1.44 (−41.1%)
1f	7.07	5.95	−1.12 (−15.8%)
1g	6.81	2.91	−3.90 (−57.3%)
1h	1.74	0.83	−0.91 (−52.3%)
1i	4.58	3.20	−1.38 (−30.1%)
1j	2.27	1.16	−1.11 (−48.9%)

**Table 6:** Adjusted wound area measured by a mobile application and the final errors reported.

Figure	Adjusted Wound Area Measured by a Mobile Application (cm^2^)	Difference (cm^2^)
1a	3.64	−0.28 (−7.1%)
1b	8.76	+0.20 (+2.3%)
1c	9.01	+1.49 (+19.8%)
1d	3.39	−1.59 (−31.9%)
1e	3.42	−0.08 (−2.3%)
1f	8.01	+0.94 (+13.3%)
1g	3.92	−2.98 (−43.8%)
1h	1.12	−0.62 (−35.6%)
1i	4.31	−0.27 (−5.9%)
1j	1.56	−0.71 (−31.3%)

## Data Availability

Not applicable.
